# Sustainable Synthesis of Adipic Acid via MnO_x_-Catalyzed Electrooxidation of Cyclohexanol in Neutral Electrolyte

**DOI:** 10.3390/molecules30142937

**Published:** 2025-07-11

**Authors:** Jiaming Shi, Guiling Zhang, Shiying Yang, Dan Yang, Yuguang Jin, Xiaoyue Wan, Yihu Dai, Yanhui Yang, Chunmei Zhou

**Affiliations:** 1 Institute of Advanced Synthesis, School of Chemistry and Molecular Engineering, Jiangsu National Synergetic Innovation Centre for Advanced Materials, Nanjing Tech University, Nanjing 211816, China; 202361205136@njtech.edu.cn (J.S.); 202461105091@njtech.edu.cn (G.Z.); yangshiying0824@163.com (S.Y.); ias_xywan@njtech.edu.cn (X.W.); ias_yhdai@njtech.edu.cn (Y.D.); yhyang@njtech.edu.cn (Y.Y.); 2 Ordos Laboratory, Ordos 017000, China; jinyuguan@163.com

**Keywords:** manganese dioxide catalyst, cyclohexanol, electrooxidation, adipic acid, neutral solution

## Abstract

Adipic acid (AA), a pivotal precursor for nylon-6,6 and polyurethane, was synthesized via an innovative catalytic electrocatalytic oxidation strategy in this study. Four distinct MnO_x_/CNT nanocatalysts were prepared by hydrothermal and co-precipitation methods and fabricated into electrodes for the oxidation of cyclohexanol (Cy-OH) in a K_2_SO_4_ neutral solution. Comprehensive characterization revealed that the catalytic performance depended on both crystalline phase configuration and manganese valence states. MnO(OH) and MnO_x_ were identified as the main active species, with the synergy between MnO species and carbon nanotubes significantly enhancing catalytic activity. Mechanistic investigations demonstrated that under Mn^4+^-dominant conditions, low-valence manganese species facilitated Cy-OH-to-cyclohexanone (Cy=O) conversion, while an optimal O_ads_/O_lat_ ratio (≈1) effectively promoted subsequent Cy=O oxidation to AA. Under optimized conditions (1.25 V vs. Ag/AgCl, 80 °C, 15 h), complete Cy-OH conversion was achieved with 56.4% AA yield and exceptional Faradaic efficiency exceeding 94%. This work elucidates manganese-based electrocatalytic oxidation mechanisms, proposes a sequential reaction pathway, and establishes an environmentally benign synthesis protocol for AA, advancing sustainable industrial chemistry.

## 1. Introduction

Adipic acid (AA, C_6_H_10_O_4_), a crucial industrial dicarboxylic acid, serves as a fundamental building block in polymer manufacturing, most notably as the primary monomer for nylon-6,6 polyamide and various high-performance engineering plastics. The global market for AA is expected to reach USD 6.5 billion by 2030 [1]. Its expanding applications in emerging sectors—including biodegradable polymers (e.g., poly(butylene adipate-co-terephthalate)), thermoplastic polyurethanes, and specialty chemicals—have intensified the demand for sustainable production methods [2]. Conventional industrial routes such as nitric acid-mediated oxidation of cyclohexane [3,4], cyclohexene [5,6], or ketone-alcohol (KA) oil [7], face critical limitations: they generate stoichiometric amounts of nitrous oxide (N_2_O), a potent greenhouse gas with a global warming potential 300-fold greater than CO_2_, while concomitant NO_x_ emissions exacerbate atmospheric pollution and accelerate reactor corrosion. These environmental and operational challenges underscore the urgent need for greener AA synthesis strategies aligned with carbon-neutral objectives.

Electrocatalytic oxidation has emerged as a quite promising alternative method, which utilizes renewable electricity to drive the activation of selective carbon–hydrogen bonds (C–H) under ambient conditions. As early as 2004, Lyalin and Petrosyan reported a nickel oxyhydroxide (NiOOH) catalyst. Under a constant current of 6 mA/cm^2^, through the process of cyclohexane (CHA) dehydrogenation to form cyclohexanone (Cy=O), and then the oxidation of Cy=O to form AA, an AA yield of 11.4% was obtained. Moreover, when Cy=O was used as the substrate, an AA yield of 52.3% could be achieved [8]. Since then, Ni-based catalysts have made great progress in the development of more efficient catalysts, including NiOOH/Ni(OH)_2_/NF (Nickel Foam) [9], and Ni/Ti systems [10], demonstrating the feasibility of converting cyclohexanol (Cy-OH) to AA via sequential dehydrogenation-oxidation steps. Mechanistic studies revealed that Ni^3+^ species mediate Cy-OH dehydrogenation to Cy=O, while oxygen vacancies facilitate subsequent ketone oxidation to AA. Subsequent catalyst engineering efforts—such as Cu doping in Ni(OH)_2_ lattices [11], SDS-modified Ni(OH)_2_ interfaces [12], and vanadium-incorporated nickel layered double hydroxide [13]—achieved notable progress, with Faradaic efficiencies (FE) exceeding 90% in alkaline media (0.5–1 M NaOH/KOH). Notably, Zhao et al. attained 94.5% conversion using Fe-Mn/Mn_2_O_3_ catalysts [14], while Guo’s group achieved the electrooxidation of Cy-OH to AA at a relatively low potential; its yield rate is 0.0992 mmol h^−1^ cm^−2^, and the FE reaches 87% [15].

Despite these advances, three critical limitations persist: (1) Universal reliance on strongly alkaline electrolytes (pH > 13) raises concerns about equipment degradation and downstream neutralization costs. (2) Competing side reactions (e.g., overoxidation to succinic/glutaric acids) compromise selectivity, particularly at elevated potentials. (3) The mechanistic role of transition metal oxides in non-alkaline environments remains underexplored. To date, no studies have reported efficient electrocatalytic AA synthesis in pH-neutral media—a knowledge gap that hinders the development of corrosion-resistant, environmentally benign electrochemical systems.

**Figure 1:**
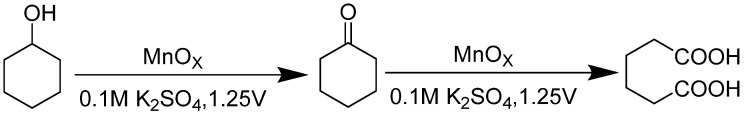
The electro-oxidation pathway of Cy-OH to AA.

Herein, we present the first manganese oxide-catalyzed electrooxidation of Cy-OH to AA in a neutral K_2_SO_4_ electrolyte (pH 7.0). Through rational design of MnO_x_/CNT hybrid catalysts, we achieve 100% Cy-OH conversion and ~60% AA selectivity at 1.25 V vs. Ag/AgCl without alkaline additives or sacrificial oxidants. Operando IR spectroscopic analyses and DFT calculations elucidate a dual-active-site mechanism: Mn^3+^ centers mediate alcohol dehydrogenation, while adjacent Mn^4+^-O-Mn^3+^ interfaces promote ketone oxidative cleavage. This work establishes a paradigm for the transition of metal oxide-catalyzed C–C bond scission in neutral aqueous media, offering a scalable route to sustainable AA production with reduced carbon footprint. Figure 1 shows the electro-oxidation pathway of Cy-OH to AA in this work.

## 2. Results and Discussion

### 2.1. Structural Properties of the Catalysts

**Figure 2:**
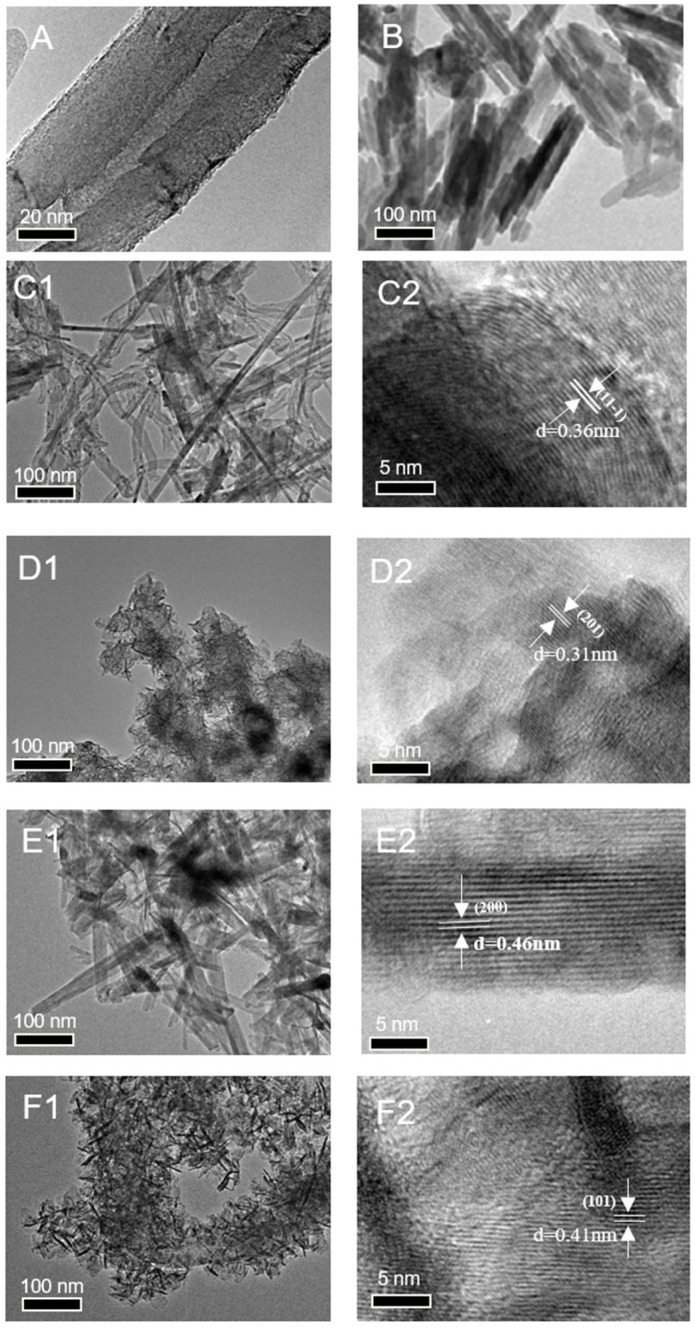
TEM and HRTEM images of CNT (**A**), MnO_x_-NR (**B**), MnO_x_/CNT-A1 (**C1**,**C2**), MnO_x_/CNT-A2 (**D1**,**D2**), MnO_x_/CNT-S (**E1**,**E2**), and MnO_x_/CNT-C (**F1**,**F2**).

As shown in Figure 2A,B, the CNT substrate exhibits a characteristic tubular morphology with well-defined structural integrity, and the commercial manganese dioxide (MnO_x_-NR) appears in a short rod shape. TEM characterization of MnO_x_/CNT-A1 and MnO_x_/CNT-S catalysts (Figure 2(C1,E1)) reveals the conformal deposition of two-dimensional MnO_2_ nanosheets uniformly coating the CNT surfaces. High-resolution TEM (HRTEM) analysis (Figure 2(C2,E2)) demonstrates distinct lattice fringes with measured d-spacings of 0.36 nm and 0.46 nm, corresponding to the (11-1) crystallographic plane of MnOOH (PDF#41-1379) and the (200) plane of γ-MnO_2_ (PDF#44-0142), respectively. In contrast, the MnO_x_/CNT-A2 and MnO_x_/CNT-C composites exhibit markedly different growth patterns (Figure 2(D1,F1)). The MnO_x_ nanostructures display three-dimensional dendritic growth along the CNT axis, forming dense surface coatings with short needle-like protrusions (50–80 nm in length). Furthermore, elemental mapping analysis of the MnO_x_/CNT-C catalyst (Figure S1) corroborates the TEM observations, demonstrating the uniform dispersion of MnO_x_ nanoparticles over the carbon nanotube (CNT) support surface.

Comparative HRTEM examination (Figure 2(D2,F2)) reveals relatively diffuse lattice fringes in these catalysts, suggesting lower crystallinity compared to their sheet-type counterparts. The attenuated fringe contrast and broader diffraction maxima indicate a predominant amorphous phase configuration containing structural defects (e.g., oxygen vacancies and lattice distortions), which may contribute to enhanced catalytic activity through increased active site density. Lattice parameter analysis confirms the presence of orthorhombic MnO_2_ phases, with measured d-spacings of 0.31 nm and 0.41 nm corresponding to the (201) and (101) planes (PDF#44-0142), respectively.

**Figure 3:**
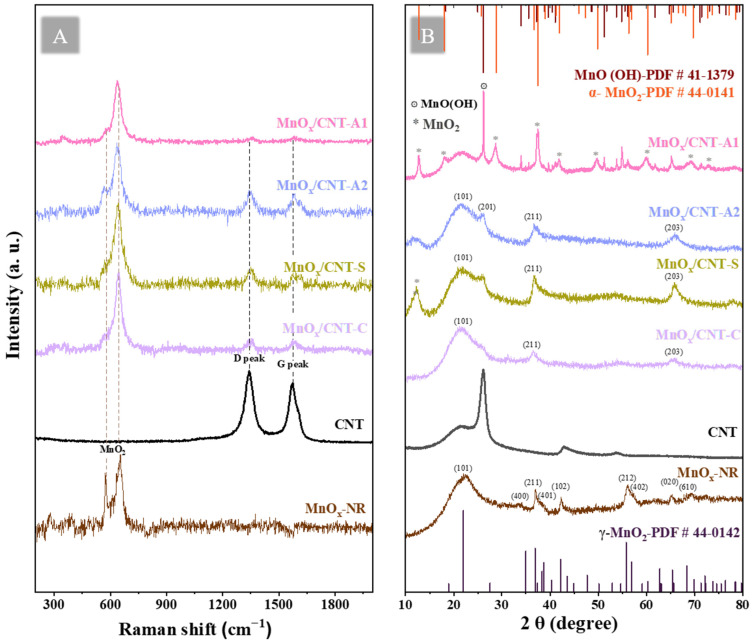
(**A**) Raman and (**B**) XRD patterns of MnO_x_/CNT-A1, MnO_x_/CNT-A2, MnO_x_/CNT-S, MnO_x_/CNT-C, CNT, and MnO_x_-NR.

As illustrated in Raman patterns of Figure 3A, the CNT matrix exhibits characteristic D and G bands at 1339.58 cm^−1^ and 1571.81 cm^−1^ respectively, corresponding to disordered to sp^3^ carbon and graphitic sp^2^ carbon vibrations [16]. All MnO_x_-based composites (MnO_x_-NR, MnO_x_/CNT-A1, MnO_x_/CNT-A2, MnO_x_/CNT-S, and MnO_x_/CNT-C) display two distinct phonon modes at 574 cm^−1^ and 641 cm^−1^, characteristic of cryptomelane-type MnO_2_ with 2 × 2 tunnel structures. These modes are attributed to the Mn-O stretching vibration and symmetric stretching of MnO_6_ octahedra, respectively [17]. Notably, the well-defined peak at 641 cm^−1^ confirms the formation of tetragonal α-MnO_2_ with high crystallinity. Upon deposition onto CNT surfaces, the Mn-O stretching intensity undergoes significant attenuation, particularly for MnO_x_/CNT-A1 (ΔI ≈ 78%), suggesting optimized interfacial interactions that suppress lattice vibrations. Concurrently, the CNT-derived D/G bands at 1344.35 cm^−1^ and 1374.72 cm^−1^ exhibit intensity reductions, reveals CNT was encapsulated by manganese dioxide and further evidencing charge transfer between CNT and MnO_x_ phases.

XRD patterns in Figure 3B confirm effective MnO_x_ coating on CNT, as evidenced by the absence of graphitic (002) reflections at ~26°. The hydrothermally synthesized MnO_x_/CNT-A1 demonstrates enhanced crystallinity relative to co-precipitated counterparts (MnO_x_/CNT-A2/S/C), manifested through sharper (211) and (203) diffraction peaks at 42.2° and 65.7° (2θ). Rietveld refinement identifies MnO(OH) (PDF # 41-1379) and α-MnO_2_ (PDF # 44-0141) as primary phases in MnO_x_/CNT-A1. Comparatively, co-precipitated composites exhibit structural similarity to MnO_x_-NR, with dominant (101), (211), and (203) planes at 22.0°, 42.2°, and 65.7° (2θ). The reduced peak intensity in MnO_x_/CNT-C (FWHM increase ~34% vs. MnO_x_-NR) implies CNT-induced lattice strain during heterogeneous nucleation, consistent with TEM-observed interfacial bonding (Figure 2(D1,D2)). This synergistic CNT-MnO_x_ interaction facilitates electron transport pathways, as corroborated by enhanced cyclohexanol electrooxidation activity (see Section 2.2).

**Figure 4:**
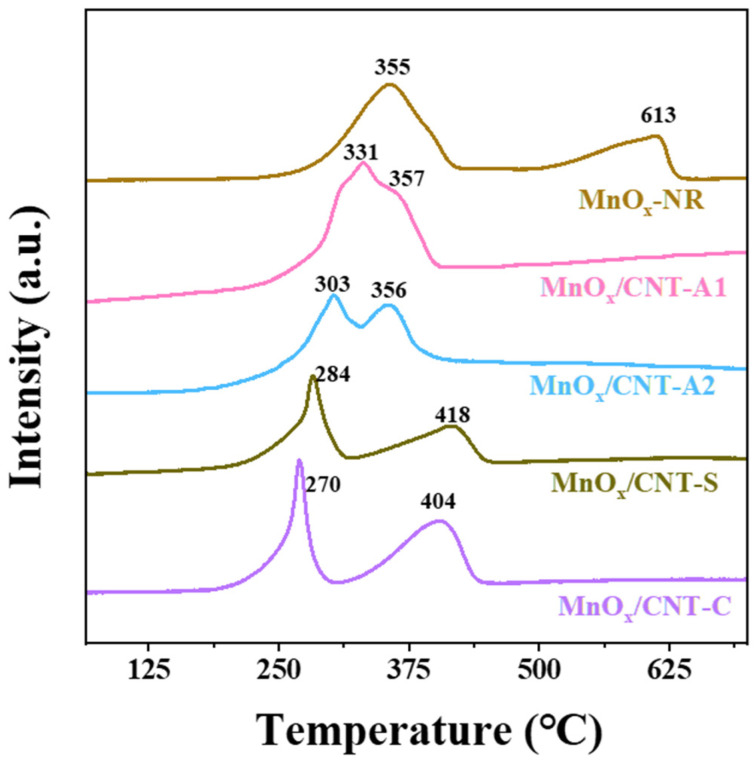
H_2_-TPR profiles of MnO_x_-NR, MnO_x_/CNT-A1, MnO_x_/CNT-A2, MnO_x_/CNT-S, and MnO_x_/CNT-C.

The H_2_-TPR profiles presented in Figure 4 reveal that all catalysts exhibit two distinct reduction peaks within the temperature range of 150–630 °C, corresponding to the stepwise reduction process: MnO_2_ → Mn_2_O_3_ (or Mn_3_O_4_) → MnO [18]. While MnO_x_/CNT-A1 and MnO_x_/CNT-A2 demonstrate overlapping reduction peaks, the remaining three catalysts (MnO_x_/CNT-S, MnO_x_/CNT-C, and MnO_x_-NR) display analogous peak configurations, suggesting comparable reduction capabilities among these materials. Notably, the first reduction peaks of the synthesized catalysts appear at lower temperatures compared to commercial manganese dioxide (MnO_x_-NR), indicating enhanced oxidation capacity, especially MnO_x_/CNT-C sample with lowest peak temperature appears at 270 °C. Furthermore, the second reduction peaks of MnO_x_/CNT-S, MnO_x_/CNT-C and MnO_x_-NR appear at higher temperatures, suggesting that their Mn^3+^ species exhibit greater resistance to reduction to Mn^2+^. This thermal stability difference implies stronger metal-support interactions in the synthesized catalysts.

**Figure 5:**
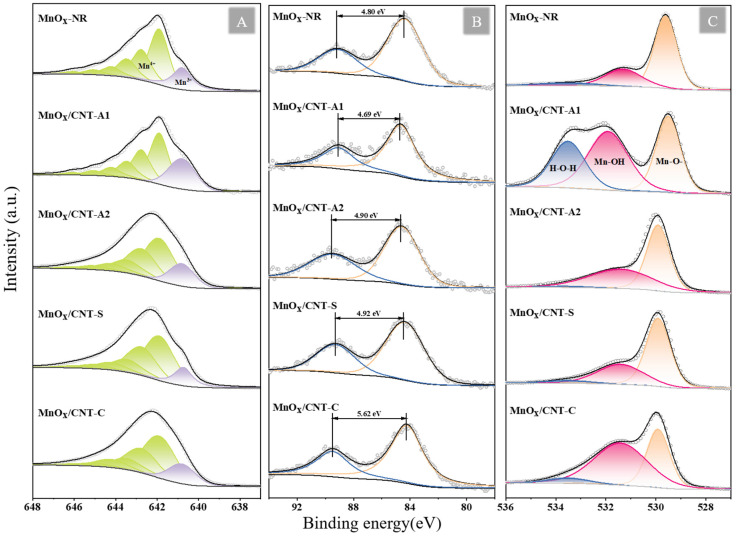
XPS spectra of MnO_x_-NR, MnO_x_/CNT-A1, MnO_x_/CNT-A2, MnO_x_/CNT-S, and MnO_x_/CNT-C at (**A**) Mn 2p, (**B**) Mn 3s, and (**C**) O 1s orbitals.

Figure 5 presents the XPS spectra of Mn 2p, Mn 3s, and O 1s core levels for the investigated catalysts. Deconvolution analysis of Mn 2p spectra (Figure 5A) demonstrates six characteristic Mn^4+^ multiplet splitting features accompanied by a distinct Mn^3+^ satellite peak centered at 640.8 eV across all catalyst systems [19]. Notably, prior mechanistic investigations have identified the oxidation of Mn^3+^ to Mn^4+^ as the rate-determining step in manganese-based catalytic cycles, highlighting the necessity of maintaining an optimal Mn^3+^/Mn^4+^ redox equilibrium for enhanced catalytic efficiency [20]. In Figure 5B, the Mn 3s splitting phenomenon exhibits characteristic energy separation (ΔE) that correlates with manganese oxidation states. A systematic comparison reveals ΔE values of 5.62 eV for MnO_x_/CNT-C and 4.69 eV for MnO_x_/CNT-A1, corresponding to higher and lower average manganese oxidation states, respectively. This disparity in ΔE values (ΔE = 0.93 eV) suggests substantial variations in metal-support interactions and electronic structure modulation induced by different synthetic protocols [21]. In Figure 5C, O 1s spectral deconvolution identifies three distinct oxygen species: lattice oxygen (O_lat_, 529.9 eV), adsorbed oxygen (O_ads_, 531.4 eV), and surface hydroxyl groups (533.5 eV) in MnO_x_/CNT-A2, –S, and –C catalysts. Intriguingly, the MnO_x_/CNT-A1 catalyst exhibits a 0.4 eV negative shift in O_lat_ binding energy (529.5 eV), indicative of increased low-valent manganese content. This observation aligns with established correlations between oxygen vacancy concentration and O_ads_/O_lat_ ratios [9]. Comparative analysis reveals that MnO_x_ incorporation significantly enhances O_ads_/O_lat_ ratios across catalysts. The corresponding electronic structure parameters are systematically compiled in Table 1 to facilitate the comparative analysis of catalytic performance.

### 2.2. Electro-Catalytic Performance

**Figure 6:**
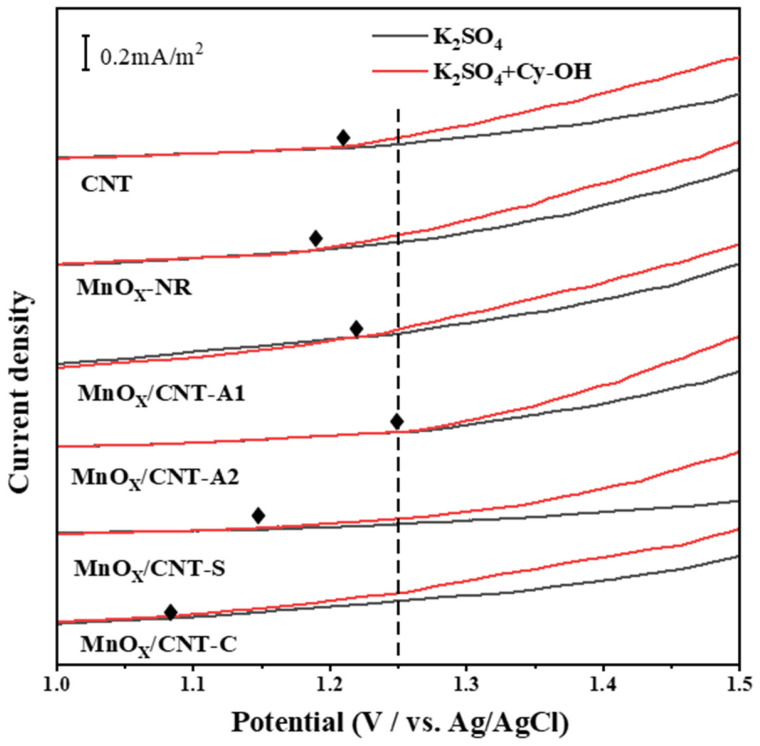
LSV profiles of electrocatalytic oxidation of Cy-OH obtained by different catalysts. Reaction conditions: 25 °C, 5 mg catalysts, 30 mL 0.1 M K_2_SO_4_ with or without 80 mg Cy-OH, 50 mV/s.

Cyclic voltammetry (CV) measurements were performed in K_2_SO_4_ electrolyte to evaluate the electrocatalytic activity of various catalysts towards cyclohexanol (Cy-OH) oxidation (Figure S2). After the addition of Cy-OH, due to the electrochemical oxidation of cyclohexanol, the onset potential is significantly reduced, and the current density is increased. For the bare electrode and CNT, although there is an increase in current, it is much lower than that of the MnO_x_-based composites, which proves that MnO_x_ is the key active component for catalyzing the oxidation of Cy-OH. Although variations in background current and capacitance were observed among the catalysts, distinct oxidation peaks corresponding to Cy-OH emerged at potentials exceeding 1.0 V (vs. Ag/AgCl) for all catalysts. For clearer comparison, linear sweep voltammetry (LSV) curves within the potential range of 1.0 to 1.5 V were extracted and analyzed (Figure 6). Compared to the baseline system without Cy-OH substrate, all catalytic systems exhibited significantly enhanced current responses in their LSV curves when the applied potential exceeded 1.25 V. The observed current density increase in the Cy-OH-free system within this potential window can be attributed to water electrolysis, while the distinct potential-dependent behavior of the Cy-OH-containing systems suggests that substrate introduction effectively enhances electrochemical reaction kinetics, providing indirect evidence for oxidative conversion of Cy-OH at the electrode surface. Notably, MnO_x_/CNT-C catalyst demonstrates a 170 mV negative shift in oxidation onset potential (1.08 V) compared to MnO_x_/CNT-A1 (1.25 V), indicating superior catalytic activity towards Cy-OH oxidation. As a critical parameter for evaluating electrocatalytic performance, current density directly reflects reaction kinetics at the electrode interface. At the characteristic potential of 1.25 V, MnO_x_/CNT-C achieves a peak current density of 0.02 mA·cm^−2^, which substantially exceeds those of other comparative catalysts, further confirming its optimal catalytic efficiency.

**Figure 7:**
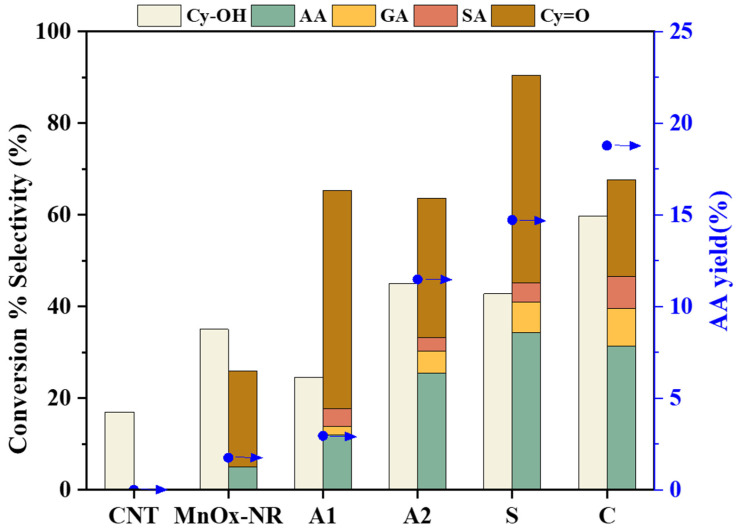
Catalytic performance of CNT, MnO_x_-NR, MnO_x_/CNT-A1, MnO_x_/CNT-A2, MnO_x_/CNT-S, and MnO_x_/CNT-C (succinic acid (SA), glutaric acid (GA)). (Reaction conditions: 20 mg catalysts, 1.25 V, 80 °C, and 60 mL 0.05 M K_2_SO_4_ with 0.8 mmol Cy-OH, 24 h).

Figure 7 presents the catalytic performance of different catalysts for the electrochemical oxidation of Cy-OH. The CNT-only system exhibited no product formation, though approximately 20% substrate consumption was observed, attributed to voltage-induced polymerization. MnO_x_-NR demonstrated limited catalytic activity with 33.9% Cy-OH conversion and only 1.7% AA yield, accompanied by 7% cyclohexanone (Cy=O) formation as an intermediate. In contrast, supported catalysts showed substantially enhanced performance. Notably, the MnO_x_/CNT-C catalyst achieved 59.8% Cy-OH conversion and 18.8% AA yield after 24 h reaction, representing optimal catalytic efficiency. These results confirm that MnO_x_ supported on CNT significantly enhances the selective oxidation of Cy-OH to AA.

The catalytic performance varied significantly with synthesis methodology. Catalysts prepared via co-precipitation (A2, S, and C) exhibited superior conversion rates and AA selectivity compared to the hydrothermal-derived A1 catalyst (140 °C), which showed limited activity (25% conversion). This discrepancy may originate from structural differences: the hydrothermal method produced catalysts with well-crystallized structures and fewer surface defects, potentially reducing active site availability. The enhanced performance of supported catalysts suggests that synergistic interactions between CNT and MnO_x_ components facilitate electron transfer processes, effectively directing the reaction pathway toward AA production.

### 2.3. Exploration of Reaction Mechanism

**Figure 8:**
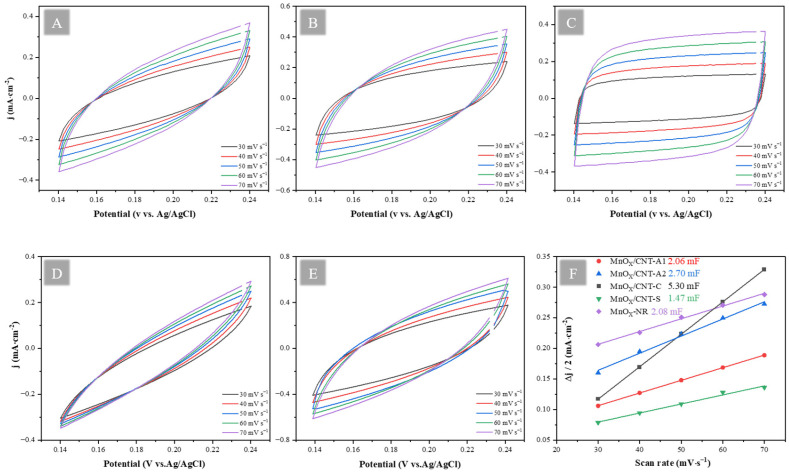
CV curves of MnO_x_-A1 (**A**), MnO_x_/CNT-A2 (**B**), MnO_x_/CNT-C (**C**), MnO_x_/CNT-S (**D**), and MnO_x_-NR (**E**) in the potential range of 0.14–0.24 V (vs. Ag/AgCl) and linear fitting (**F**) of the capacitive currents and scan rates for the different catalysts.

From the product distribution profile depicted in Figure 7, the oxidative transformation pathway of Cy-OH can be mechanistically proposed. The predominant reaction pathway proceeds through sequential oxidation steps is as follows: initial conversion of Cy-OH to Cy=O), followed by subsequent oxidation to AA. A competitive side pathway involves over-oxidation and carbon chain fragmentation of AA, yielding shorter-chain dicarboxylic acids including glutaric acid (GA) and succinic acid (SA). To elucidate this mechanism, we conducted systematic kinetic measurements of the consecutive oxidation steps (Cy-OH → Cy=O and Cy=O → AA) using respective substrates over various catalysts (Table 1). The experimental data reveal that all supported catalysts (except MnO_x_-NR with notably diminished activity due to its low specific surface area of 19.92 m^2^/g) maintained initial reaction rates within 50~200 mmol·g^−1^·min^−1^. Through comparative analysis of surface electronic configurations, MnO_x_/CNT-C with the highest Mn^3+^/Mn^4+^ ratio (0.18) and lowest average oxidation state (AOS = 3.03) demonstrated superior catalytic activity (r_1_ = 153.07 mmol·g^−1^·min^−1^) in the primary oxidation step [22]. Additionally, the electrochemical behavior of each catalyst was characterized by cyclic voltammetry (CV), and the active sites and electrochemical activity of different catalysts were quantitatively analyzed based on the double-layer capacitance (Cdl). Figure 8 shows that the Cdl value of MnO_x_/CNT-C is 5.30 mF, which is significantly higher than that of other samples. This data difference further indicates that MnO_x_/CNT-C has richer active sites, which can accelerate the transfer of β-hydrogen and the release of protons in the dehydrogenation reaction, while promoting the rapid transfer of electrons to the electrode to maintain the regeneration of active sites (O=Mn^3+^-H → O=Mn^3+^-H). In contrast, low double-layer capacitance, such as that of MnO_x_/CNT-S, results in weak charge storage capacity, leading to intermediate accumulation and slow reaction. By optimizing the double-layer capacitance, the catalytic cycle efficiency can be improved, thereby increasing the generation rate and selectivity of target products such as carboxylic acids.

In the secondary oxidation stage, MnO_x_/CNT-A2 and MnO_x_/CNT-S catalysts with balanced adsorbed-to-lattice oxygen ratios (O_ads_/O_lat_ ≈ 1) achieved enhanced reaction rates of 169.74 and 147.39 mmol·g^−1^·min^−1^, respectively. This observation aligns with Chen et al.’s findings, emphasizing the critical role of metal valence states in catalytic performance [23]. Detailed correlation analysis between electronic parameters and catalytic activity (Table 1) demonstrates that Mn^3+^-enriched catalysts with elevated Mn^3+^/Mn^4+^ ratios in Mn^4+^-predominant systems exhibit superior oxidative capability. The inferior performance of MnO_x_/CNT-A1 can be attributed to its high crystallinity, reduced defect density, and limited active site accessibility. The optimal O_ads_/O_lat_ ratio ≈ 1 corroborates the Mars-van Krevelen (MvK) mechanism involving lattice oxygen participation in redox cycles [24]. These findings collectively establish that synergistic coordination between adsorbed and lattice oxygen species is crucial for achieving efficient Cy=O to AA conversion kinetics.

**Figure 9:**
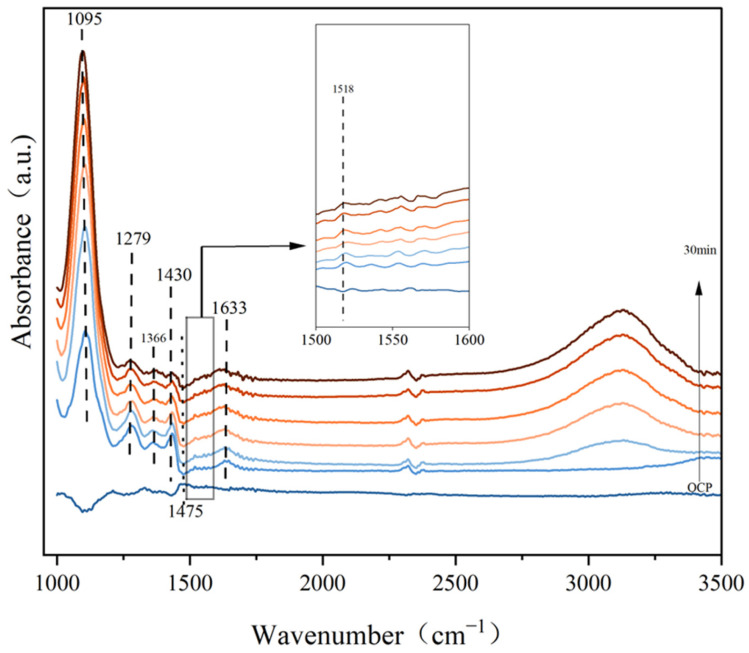
Time-resolved in situ FTIR spectra documenting Cy-OH electrooxidation over MnO_x_/CNT-C. (Experimental parameters: 2 mg catalyst, 0.8 mmol Cy-OH in 60 mL 0.1 M K_2_SO_4_, 80 °C, 0–30 min at 1.25 V).

In situ electrochemical FTIR was employed to monitor the dynamic evolution of surface intermediates during the reaction (Figure 9). Spectral acquisition was conducted in 0.1 M K_2_SO_4_ containing Cy-OH at 1.25 V applied potential over 30 min. The observed distinct enhancement bands at 1095 cm^−1^ (C–O stretching), 1279 cm^−1^ (C–C skeletal vibration), and 1633 cm^−1^ (C=O stretching) were associated with the formation and subsequent consumption of Cy=O. Emerging characteristic peaks at 1430 cm^−1^ (O–H bending) and 1518 cm^−1^ (C=O stretching) corresponded to the generation of AA species. Notably, the relatively weak peak intensities suggest limited AA production efficiency under these reaction conditions. A prominent inverted absorption band at 1475 cm^−1^ (C–H deformation) [11,15,24] indicates substantial Cy-OH depletion. The enhanced inversion depth observed in this system implies accelerated Cy-OH consumption kinetics, potentially attributable to improved surface reactivity of the MnO_x_ catalyst.

**Scheme 1:**
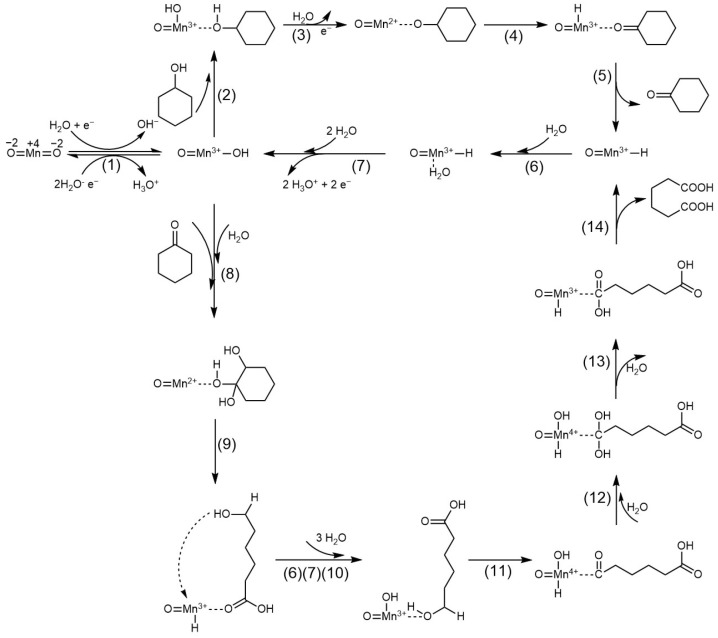
Proposed dual catalytic cycles for (top) Cy-OH dehydrogenation to Cy=O and (bottom) Cy=O oxidative cleavage to AA over MnO_x_/CNT interfaces.

Based on established mechanistic frameworks for Ni-based catalyst [9,11] and the mechanism of RuO_2_ electrocatalytic alcohol oxidation [25], a dual-stage catalytic cycle is proposed for the electrocatalytic oxidation of Cy-OH to AA over MnOx catalysts (Scheme 1). In the initial phase, the Mn^4+^ catalytic center accepts an electron (e^−^) from a water molecule in the electrolyte and combines with OH^−^ to form a surface hydroxylated species O=Mn^3+^–OH (Step 1). Cyclohexanol (Cy-OH) coordinates via its hydroxyl group (–OH) to the Mn^3+^ site, forming an adsorbed state that initiates dehydrogenation (Step 2). Subsequent cleavage of the O–H bond in adsorbed cyclohexanol releases a proton (H^+^), which combines with the –OH group in O=Mn^3+^–OH to form and liberate a water molecule. Concurrently, O=Mn^3+^–OH is oxidized to an O=Mn^2+^ species bound to the dehydrogenation intermediate (Step 3). The α-H of this intermediate transfers to the O=Mn^2+^ site, generating O=Mn^3+^–H and cyclohexanone (Cy=O) (Steps 4–5). Finally, O=Mn^3+^–H undergoes hydrolysis with three water molecules to regenerate the active center O=Mn^3+^–OH (Steps 6–7) [26].

In the secondary stage, cyclohexanone first adsorbs to the O=Mn^3+^–OH site and combines with H_2_O to form a geminal diol (Step 8) [26]. Simultaneously, the α-hydrogen (α-H) of cyclohexanone bonds to oxygen to generate a hydroxyl group, consuming the hydroxyl group in O=Mn^3+^–OH and reducing it to an O=Mn^2+^ site (Step 8) [27]. In the presence of the geminal diol, C–C bond cleavage occurs synchronously with carboxyl and alcohol group formation (Step 9) [28]; concurrently, one hydrogen from the geminal diol transfers to the Mn site forming O=Mn^3+^–H (Step 9). O=Mn^3+^–H regenerates to O=Mn^3+^–OH via the mechanism in Steps 6–7. This site further adsorbs the alcohol group at the opposite terminus of the intermediate, yielding the Step 10 intermediate. This intermediate undergoes dehydrogenation analogous to Steps 3–4: alcohol group oxidation to aldehyde (Step 11), aldehyde hydration to geminal diol (Step 12), and dehydration to carboxyl group (Step 13). Ultimately, adipic acid (AA) desorbs, leaving an O=Mn^3+^–H site (Step 14). During Steps 11–13, the secondary hydroxyl group (–CH(OH)–) in the linear molecule is dehydrogenated via a proton-coupled electron transfer (PCET) mechanism at the Mn^3+^ site to form a carbonyl group (–C=O–), which is further oxidized to carboxyl (–COOH). O=Mn^3+^–H regenerates to the initial O=Mn^3+^–OH (or MnO_2_) state via Steps 6, 7, and 1, completing the catalytic cycle from cyclohexanone to adipic acid.

### 2.4. Optimization of Reaction Conditions

**Figure 10:**
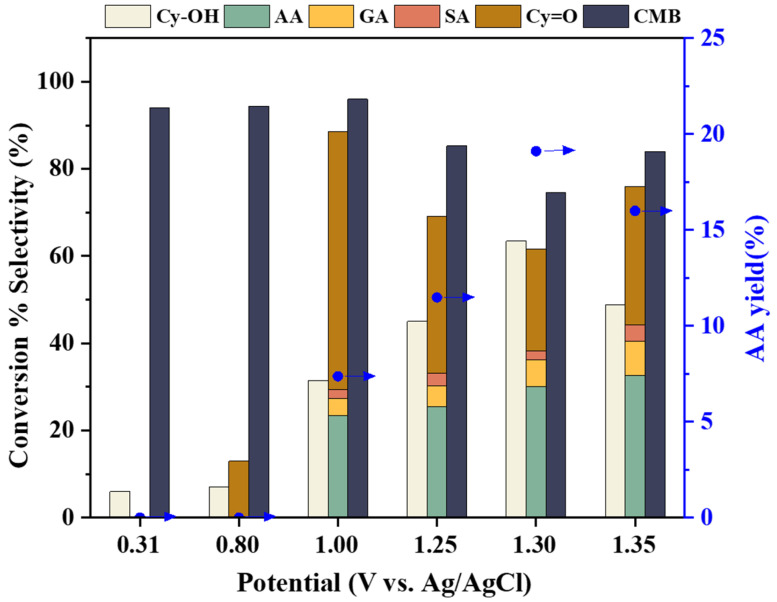
Catalytic performance of electro-oxidation Cy-OH with different potential. (Reaction conditions: 20 mg MnO_x_/CNT-A2, 0.8 mmol Cy-OH, 60 mL 0.05 M K_2_SO_4_, 80 °C, 24 h).

The LSV profiles in Figure 6 reveal that most of the catalysts exhibit a characteristic oxidation peak of Cy-OH at a potential of approximately 1.15 V (vs. Ag/AgCl). Therefore, taking catalyst MnO_x_-CNT-A2 as an example, systematic potentiostatic electrolysis experiments (Figure 10) were conducted to investigate the electrochemical oxidation behavior under applied potentials of 0.31 V (open circuit potential, OCP), 0.8 V, 1.0 V, 1.25 V, 1.3 V, and 1.35 V. Experimental results demonstrate that no detectable products were formed at OCP (0.31 V), while both Cy-OH conversion and AA yield showed significant enhancement with applied potential increasing from 0.8 to 1.3 V. Kinetic analysis reveals a non-monotonic dependence of Cy-OH conversion on potential, initially increasing then decreasing, whereas AA selectivity stabilized after reaching a plateau value. This confirms the critical role of working potential in regulating substrate conversion. Notably, at the optimized potential of 1.25 V, the system achieved superior synergistic performance: Cy-OH conversion exceeded 40% with concomitant AA yield of 12% and selectivity of 25%. When the potential increased to 1.30 V, although Cy-OH conversion improved to ~60%, the carbon mass balance significantly decreased from 90% to 75%, indicating the activation of parasitic reaction pathways. Further elevation to 1.35 V unexpectedly reduced Cy-OH conversion to 50%, likely due to competing oxygen evolution reactions at the catalyst surface under excessive potentials. Comprehensive evaluation suggests that 1.25 V represents the optimal working potential, effectively balancing reaction efficiency with suppression of side reactions in this electrocatalytic system.

**Figure 11:**
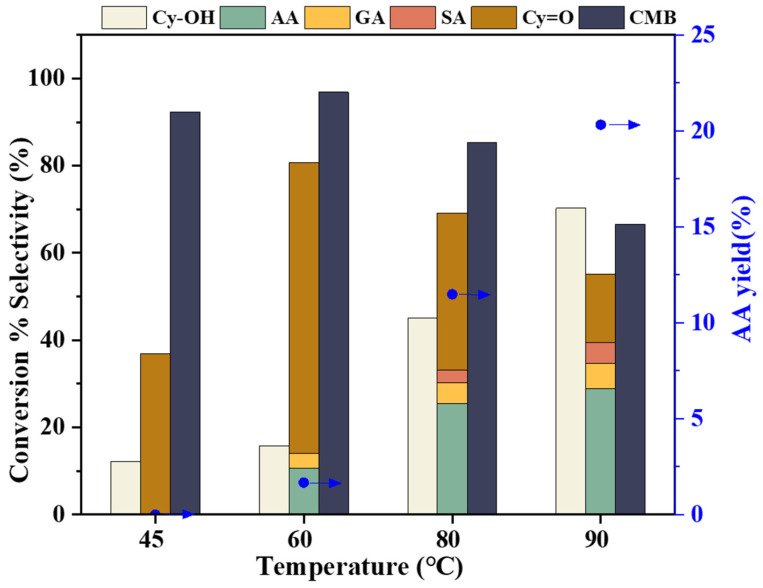
Catalytic performance of Cy-OH electro-oxidation with different temperatures. (Reaction conditions: 20 mg MnO_x_/CNT-A2, 0.8 mol Cy-OH, 60 mL 0.05 M K_2_SO_4_, 1.25 V, 24 h).

The temperature-dependent electrochemical oxidation performance of Cy-OH over MnO_x_/CNT-A2 catalyst was systematically investigated at 1.25 V applied potential across four temperatures (45 °C, 60 °C, 80 °C, and 90 °C), as illustrated in Figure 11. Initial experiments revealed limited conversion efficiencies of 12.1% and 15.8% at 45 °C and 60 °C, respectively. Notably, the target product AA first emerged at 60 °C, though with a modest yield of 1.7%. Both conversion efficiency and product selectivity exhibited positive temperature dependence, while the carbon balance demonstrated an inverse correlation with temperature elevation. This carbon loss principally originated from two pathways: (1) enhanced formation of dicarboxylic acid byproducts (SA and GA), and (2) concentration-driven oligomerization/polymerization of reaction products. At 90 °C, despite achieving a substantial conversion rate of 70.3%, the carbon balance significantly deteriorated to 66.6%, likely attributable to thermal cleavage of C–C bonds. Comprehensive analysis of the data suggests 80 °C as the optimal reaction temperature, balancing a 45% conversion rate with 11.5% AA yield while maintaining 85.3% carbon balance.

**Figure 12:**
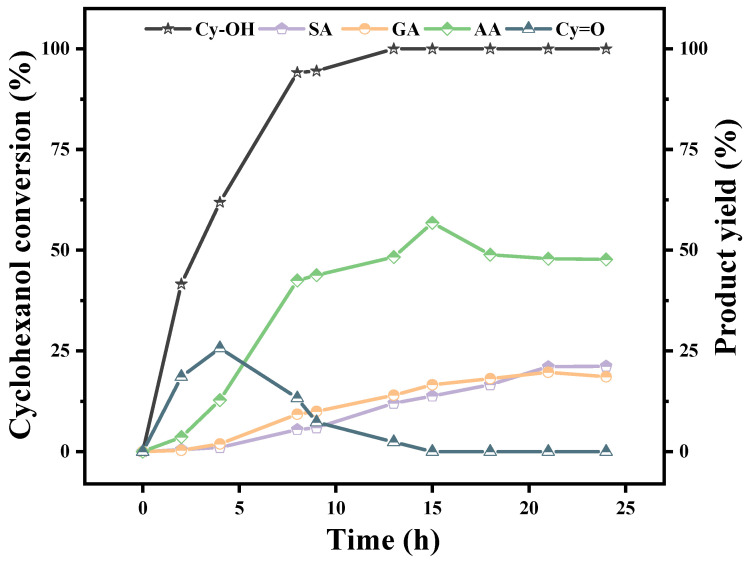
The percentage conversion of Cy-OH and yield of products with different reaction time. (Reaction conditions: 40 mg MnO_x_/CNT-C, 0.8 mmol Cy-OH, 30 mL 0.1 M K_2_SO_4_, 1.25 V, 80 °C).

Figure 12 illustrates the temporal evolution of Cy-OH conversion and product distribution under optimized conditions (80 °C, 1.25 V, 40 mg catalyst). The Cy-OH conversion exhibited a monotonic increase until complete substrate depletion. Notably, approximately 95% of Cy-OH was rapidly consumed within the initial 9 h period. The intermediate product cyclohexanone (Cy=O) displayed characteristic volcano-type behavior, reaching maximum yield before subsequent depletion to form AA, SA, or GA. The target product AA achieved peak yield (56.4%) at 15 h reaction time, followed by a gradual decline potentially attributed to concentration-dependent polymerization phenomena. Mechanistic analysis suggests an eight-electron transfer process during the reaction. The calculated FE reached 94.5% at the 15 h timepoint.

After the completion of the catalytic reaction, the morphology of the catalyst was characterized. The TEM and HRTEM images (Figure S3) reveal that the overall morphology and dispersion of MnO_X_ nanoparticles on the CNT support remain largely unchanged after the reaction, indicating good structural stability under the applied electrochemical conditions. Figure S4 shows the XPS spectra of Mn 2p, Mn 3s, and O 1s core levels for the invested catalysts after the electrocatalytic oxidation reaction. Through comparison, it can be found that the peak positions of each main peak basically remain unchanged. It indicates that each catalyst remains relatively stable. Calculations (Table S1) show that Mn^3+^/Mn^4+^ has significantly increased, except for MnOX/CNT-A1. Catalysts with a higher Mn^3+^/Mn^4+^ ratio are more favorable for the alcohol oxidation stage. Each catalyst exposes more active sites, leading to an accelerated electron transfer rate and an increased reaction rate, which is beneficial for the progression of the catalytic reaction. This corresponds to the increase in their AOS (average oxidation state) values. For MnOX/CNT-A1, it may be related to its catalyst preparation method. The catalyst MnOX/CNT-A1 prepared by the hydrothermal method has a good crystalline structure and fewer surface defects, which may reduce the availability of active sites.

## 3. Experimental Section

### 3.1. Materials and Catalysts Preparation

Concentrated nitric acid treatment at 140 °C for 2 h was used for the purification of CNT (S-MWNT-2040, Shenzhen Nanometer Port Co., Ltd., Shenzhen, China) supports; KMnO_4_ (≥99.5%), HNO_3_ (69%, Sigma-Aldrich, St. Louis, MO, USA), H_2_SO_4_ (98%, Sigma-Aldrich), Citric Acid (97%, Aladdin, Shanghai, China), Mn(Ac)_2_∙4H_2_O (99%, Energy Chemical, Shanghai, China), K_2_SO_4_ (≥ACS, Aladdin), cyclohexanol (Cy-OH, ≥98%, Sinopharm Chemical Reagent Co., Ltd., Shanghai, China), cyclohexanone (Cy=O, ≥99.5%, Shanghai Lingfeng Chemical Chemical Reagent Co., Ltd., Shanghai, China), Succinic acid (≥99%, Aladdin), glutaric acid (99%, Aladdin), and adipic acid (AA, ≥99%, Aladdin) were used without further purification, and all solutions were prepared with double distilled (DI) water.

Four different catalysts (MnO_x_/CNT) were prepared by using two different precursors, KMnO_4_ and CNT. The contents of metallic manganese were controlled to be the same (the theoretical value is 67%). Only MnO_x_/CNT-A1 [29] was prepared using a hydrothermal method: 331.86 mg KMnO_4_ and 100 mg treated CNT were mixed in 60 mL DI water (solution 1), while 392.14 mg Mn(Ac)_2_∙4H_2_O leached out in 12 mL water (solution 2) under 30 min of ultra-sonication. The hydrothermal process was kept at 140 °C for 15 h when the solutions 1 and 2 were transferred to a 100 mL Teflon-lined autoclave.

The other three catalysts were prepared by the method of precipitation. A round-bottom flask, a reflux condenser and the oil bath pot are used to prepare MnO_x_/CNT-A2 [30], MnO_x_/CNT-S [31] and MnO_x_/CNT-C [32]. In the preparation of these catalysts, DI water was used as a medium, and the dosage of chemical reagents with their preparation temperatures and times are shown in Table 2. Of course, before they are heated, all mixtures should be mixed sufficiently under ultra-sonication for 30 min. Among them, MnO_x_/CNT-A2 is prepared in two steps: a given mass of Mn(Ac)_2_∙4H_2_O and CNT was used to prepare powder 1 at 80 °C for 24 h while the vapor was first condensed by a reflux condenser. Then powder 1 was used to react with KMnO_4_ in the same case after grinding. Furthermore, when preparing MnO_x_/CNT-C, citric acid solution was added dropwise via a micro flow pump; the speed was controlled to sustain the reaction process for 7 h. After the process ended, the suspensions were cooled down to room temperature. Then the powder was cleansed by DI water until PH = 7, then dried at 80 °C in oven overnight. Finally, the obtained catalysts were labeled after grinding.

### 3.2. Catalyst Characterizations

The loadings of Mn species for all catalysts were measured via an inductively coupled plasma optical emission spectrometry (ICP-OES, PerkinElmer Avio 200, Shelton, CT, USA). The loading of MnO_x_/CNT-A1, MnO_x_/CNT-A2, MnO_x_/CNT-S, and MnO_x_/CNT-C was all around 40%, respectively. The Raman spectroscopic information of each catalyst was collected on the Horiba HR Evolution spectrometer (Villeneuve d’Ascq, France), using 325 nm and 532 nm excitation light sources. X-ray diffraction (XRD) patterns were obtained on MiniFlex600 (Rigaku, Tokyo, Japan). The diffraction date was gathered with a 0.02-degree (2θ) resolution at a current of 20 mA and an accelerating voltage of 40 mV. The microscopic appearances of metal oxide catalyst were observed via the jeol 2100plus transmission electron microscope (TEM, Tokyo, Japan), operated at 200 kV. The surface elements and chemical forms of catalysts were recorded by X-ray photoelectron spectroscopy (XPS, Thermo ESCALAB 250XI spectrometer, Waltham, MA, USA) with Al Kα radiation. The binding energies were referenced by the C1s peak at 284.8 eV. Hydrogen temperature-programmed reduction (H_2_-TPR) was measured on a Micromeritics AutoChem 2920 chemisorption analyzer (Norcross, GA, USA). An amount of 70 mg of catalysts was purged under Ar (30 mL/min) at 120 °C for 30 min, and then reduced in 10 vol% H_2_/Ar (30 mL/min) from 50 °C to 800 °C after cooling down.

The electrochemical performances were tested on CHI 660E electrochemical workstation (Shanghai Chenhua Instruments Co., Ltd., Shanghai, China), using a three-electrode system.

### 3.3. Electrochemical Activity Testing

In this experiment, linear sweep voltammetry test and constant voltage method were used to certify the electrochemical performance of the catalysts.

The prepared catalyst, Pt plate, and Ag/AgCl electrolyte were anode, cathode and reference electrode, respectively. Cyclic voltammetry test was performed for 10 circles at a scanning speed of 50 mV/s in the 0.1 M K_2_SO_4_ solution in the presence or absence of Cy-OH, with a voltage range of 0~1.5 V (vs. Ag/AgCl).

When conducting a constant voltage test, the distance between anode and cathode remained constant. The Cy-OH oxidation was carried out in a H-shaped electrolytic cell at 80 °C. A certain amount of Cy-OH and 60 mL 0.05 M K_2_SO_4_ were added into the container. The electrocatalytic oxidation of Cy-OH occurred under the voltage 1.25 V (vs. Ag/AgCl) for 24 h. After the oxidation reaction, the substrates and products solution were diluted four times and analyzed by a high-performance liquid chromatography (Shimadzu LC-20A, Kyoto, Japan) with a Shodex SUGAR SH1011 column (8 mm I.D. × 300 mm, packed with strong acid cation exchange resin) and Refractive index detector (RID). Meanwhile, post-reaction electrolyte analysis was performed using ^1^H NMR spectroscopy. The NMR spectra (showing characteristic peaks for adipic acid and reaction intermediates) and HPLC chromatograms (quantifying adipic acid yield) are now provided in the Supporting Information (Figure S5). Excellent agreement was observed between the product identification and quantification results obtained from these two independent techniques (NMR and HPLC). Notably, HPLC analysis exhibited superior resolution for Cy-OH and Cy = O and a linear response over a wider concentration range (R^2^ > 0.999) (Figure S6). Therefore, this method was adopted for all subsequent quantitative analyses.

### 3.4. Product Analysis

(1) $$\mathrm{Con}.\;=\;\frac{n(\mathrm{Cy-OH}\;\mathrm{consumed})}{n(\mathrm{Cy-OH}\;\mathrm{initial})]}\;\times \;100\%$$

(2) $$\mathrm{Product}\;\mathrm{yield}=\frac{n\left(\mathrm{product}\;\mathrm{formed}\right)}{n\left(\mathrm{Cy-OH}\;\mathrm{initial}\right)}\;\times \;100\%$$

(3) $$\mathrm{CMB}.=\frac{6n(\mathrm{Cy-OH}\;\mathrm{remained})\cdot z\cdot n\left(\mathrm{product}\;\mathrm{formed}\right)}{6n(\mathrm{Cy-OH}\;\mathrm{initial})}\;\times \;100\%$$

(4) $$\mathrm{FE}=\frac{n(\mathrm{product}\;\mathrm{formed})\;\times \;8\;\times \;F}{I\;\times \;t}\;\times \;100\%$$

(5) $$r=\frac{1}{S}\;\times \;\frac{d\varepsilon }{\mathrm{dt}2}\;\times \;10^{-3}$$

where F is the Faraday constant (96,485 C∙mol^−1^), and n is the mol of reactant calculated from the concentration measured by HPLC. z is the number of carbon atoms in the product. I is current density (A). t is reaction time (s). r is reaction rate (mmol∙m^−2^∙h^−1^). S is specific surface area (m^2^∙g^−1^). ε is mmol of reactant conversion. t2 is time for converting Cy-OH (h).

## 4. Conclusions

In this study, four distinct manganese dioxide catalysts were successfully synthesized through hydrothermal and co-precipitation methods. The engineered electronic coupling effects between MnO_x_ and carbon nanotube (CNT) matrices demonstrated superior electrocatalytic activity for cyclohexanol oxidation in neutral aqueous media. Through comprehensive mechanistic investigations, we propose a dual-stage reaction pathway: initial dehydrogenation of Cy-OH to Cy=O, followed by oxidative cleavage to AA. Notably, catalyst systems with tetravalent manganese dominance exhibited enhanced Cy-OH to Cy=O conversion kinetics when incorporating optimal low-valent manganese sites (Mn^3+^/Mn^4+^ ratio = 0.24). Furthermore, maximized AA formation efficiency correlated with balanced surface oxygen speciation (O_ads_/O_lat_ ratio ≈ 0.82). Under optimized operational parameters (applied potential: 1.25 V vs. Ag/AgCl (0.1 M K_2_SO_4_), temperature: 80 ± 1 °C, catalyst loading: 40 mg cm^−2^, duration: 15 h), the system achieved complete substrate conversion (100%) with 56.4% AA yield and remarkable Faradaic efficiency (94.2 ± 1.8%). This electrocatalytic protocol establishes an environmentally benign alternative to conventional nitric acid oxidation processes, showing significant potential for sustainable AA production at industrial scales.

## Figures and Tables

**Table 1:** The specific surface area, reaction performance [a] and surface electronic structure of different catalysts.

Catalyst	S_BET_^[b]^ (m^2^·g^−1^)	Mn^3+^/Mn^4+^	AOS ^[c]^	O_ads_/O_lat_	r_1_^[d]^ (mmol·g^−1^·min^−1^)	r_2_^[e]^ (mmol·g^−1^·min^−1^)
MnO_X_-NR	19.96	0.07	3.55	0.40	1.93	21.66
MnO_X_/CNT-A1	49.39	0.24	3.68	1.26	58.94	107.51
MnO_X_/CNT-A2	63.06	0.10	3.43	0.82	73.89	169.74
MnO_X_/CNT-S	247.71	0.07	3.42	0.57	51.61	147.39
MnO_X_/CNT-C	166.98	0.18	3.03	1.68	153.07	120.78

[a] 20 mg catalysts, 0.8 mmol substrate, 30 mL 0.1 M K_2_SO_4_, 1.25 V (vs. Ag/AgCl), 60 °C, ~20% conversion. [b] Specific surface area calculated by BET. [c] The average oxidation state (AOS) of Mn is determined by the binding energy difference (∆Es) of the splitting peaks of Mn 3s. AOS = 8.956 − 1.126 · ΔEs [22]. [d] The reaction rate of Cy-OH to Cy=O. [e] The reaction rate of Cy=O to AA.

**Table 2:** The preparation conditions of the catalysts.

Catalyst	Chemical Regents	Temperature	Time
MnO_X_/CNT-A2	1. 250 mg CNT + 1.038 g Mn (Ac)_2_·4H_2_O + 50 mL H_2_O 2. powder 1 + 790 mg KMnO_4_ + 50 mL H_2_O	80 °C 80 °C	24 h 24 h
MnO_X_/CNT-S	133 mg CNT + 584.7 mg KMnO_4_ + 118 μL H_2_SO_4_ + 25 mL H_2_O	75 °C	1 h
MnO_X_/CNT-C	37 mL 0.05 M KMnO_4_ + 37 mL 0.05 M citric acid + 50 mg CNT	80 °C	7 h

## Data Availability

The original contributions presented in this study are included in the article/Supplementary Materials. Further inquiries can be directed to the corresponding authors.

## Supplementary Materials

The following supporting information can be downloaded at: https://www.mdpi.com/article/10.3390/molecules30142937/s1, Figure S1. TEM image of MnO_x_/CNT-C catalyst (A) and corresponding EDS elemental mapping of C (B), Mn (C), and O (D). Figure S2. Cyclic voltammograms of the electrocatalytic oxidation of Cy-OH over different catalysts. (Reaction conditions: 25 °C, 5 mg catalyst, 30 mL of 0.1 M K_2_SO_4_ with and without 0.8 mmol Cy-OH, scan rate = 50 mV s^−^^1^). Figure S3. TEM and HRTEM images of the used catalysts: MnO_x_/CNT-A1 (A), MnO_x_/CNT-A2 (B), MnO_x_/CNT-S (C), and MnO_x_/CNT-C (D). Figure S4. XPS spectra in the Mn 2p (A), Mn 3s (B), and O 1s (C) regions for used MnOx-NR, MnOx/CNT-A1, MnOx/CNT-A2, MnOx/CNT-S, and MnOx/CNT-C catalysts. Figure S5. 1H-NMR spectrum (A)and HPLC chromatogram (B) of Cy-OH, Cy=O and AA in flash and spent states. (Reaction conditions: 40 mg MnOx/CNT-C, 0.8 mmol Cy-OH, 30 mL 0.1 M K_2_SO_4_, 1.25 V, 80 °C, 4 h). Figure S6. Calibration curves (A) based on peak area versus concentration and standard peak positions (B) for Cy-OH, Cy=O, and AA in HPLC. Table S1. The surface electronic structures of different catalysts after the electrocatalytic oxidation reaction.
