# Supplementary material for: Sustainable Synthesis of Adipic Acid via MnOx-Catalyzed Electrooxidation of Cyclohexanol in Neutral Electrolyte
Source: Molecules. 2025 Jul 11;30(14):2937. doi: 10.3390/molecules30142937 (PMC12299911; doi:10.3390/molecules30142937)
Supplement: Supplementary file 1 [file molecules-30-02937-s001.zip › molecules-3640909-supplementary.pdf]

Supporting Information

Sustainable Synthesis of Adipic Acid via MnO_x-Catalyzed Electrooxidation of Cyclohexanol in Neutral Electrolyte

Jiaming Shi [1], Guiling Zhang [1], Shiying Yang [1], Dan Yang [1,*], Yuguang Jin [2], Xiaoyue Wan [1], Yihu Dai [1], Yanhui Yang [1,2] and Chunmei Zhou [1,*]

[1] Institute of Advanced Synthesis, School of Chemistry and Molecular Engineering, Jiangsu National Synergetic Innovation Centre for Advanced Materials, Nanjing Tech University, Nanjing 211816, China

[2] Ordos Laboratory, Ordos 017000, China

***** Correspondence: yangdan@njtech.edu.cn; ias_cmzhou@njtech.edu.cn

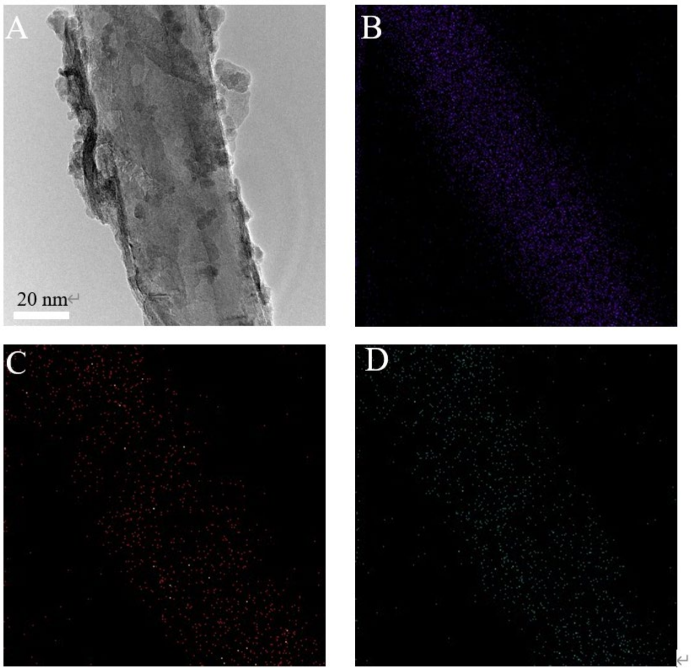

Figure S1. TEM image of MnO_x/CNT-C catalyst (A) and corresponding EDS elemental mapping of C (B), Mn (C), and O (D).

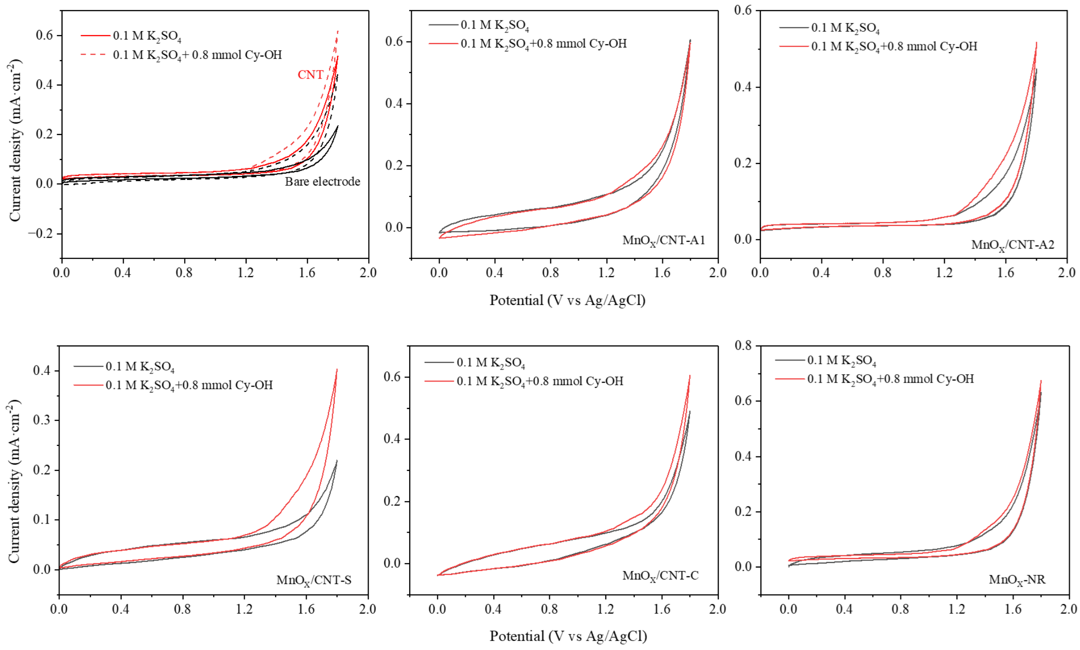

Figure S2. Cyclic voltammograms of the electrocatalytic oxidation of Cy-OH over different catalysts. (Reaction conditions: 25 °C, 5 mg catalyst, 30 mL of 0.1 M K_2SO_4 with and without 0.8 mmol Cy-OH, scan rate = 50 mV s^{-1}.)

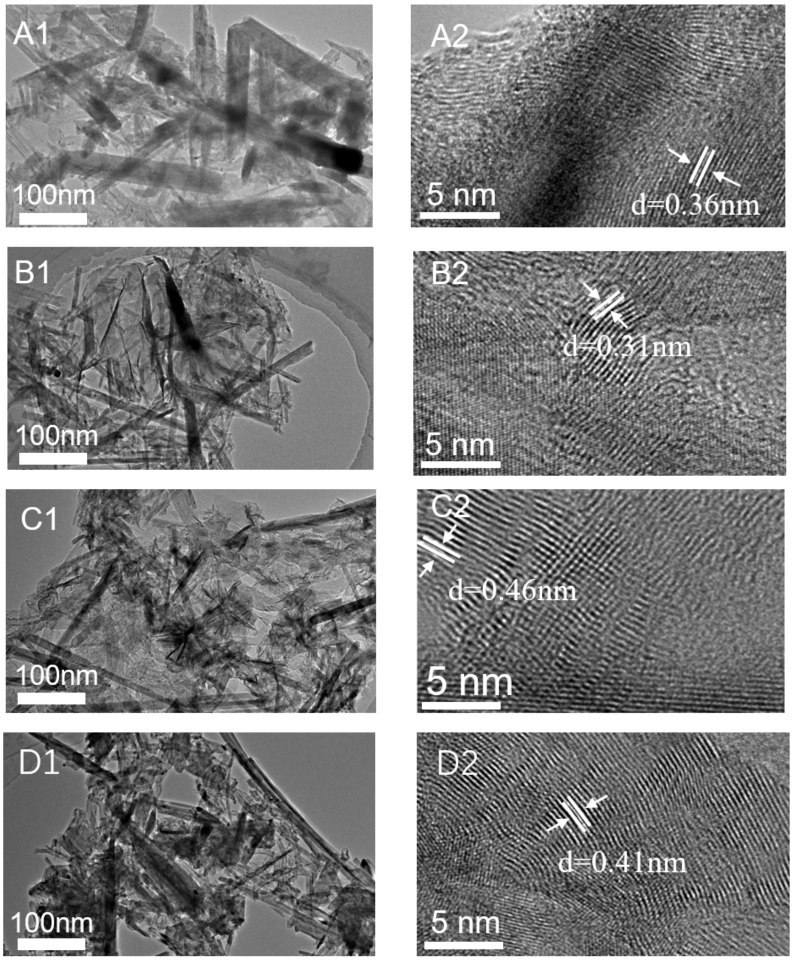

Figure S3. TEM and HRTEM images of the used catalysts: MnO_x/CNT-A1 (A), MnO_x/CNT-A2 (B),MnO_x/CNT-S (C), and MnO_x/CNT-C (D).

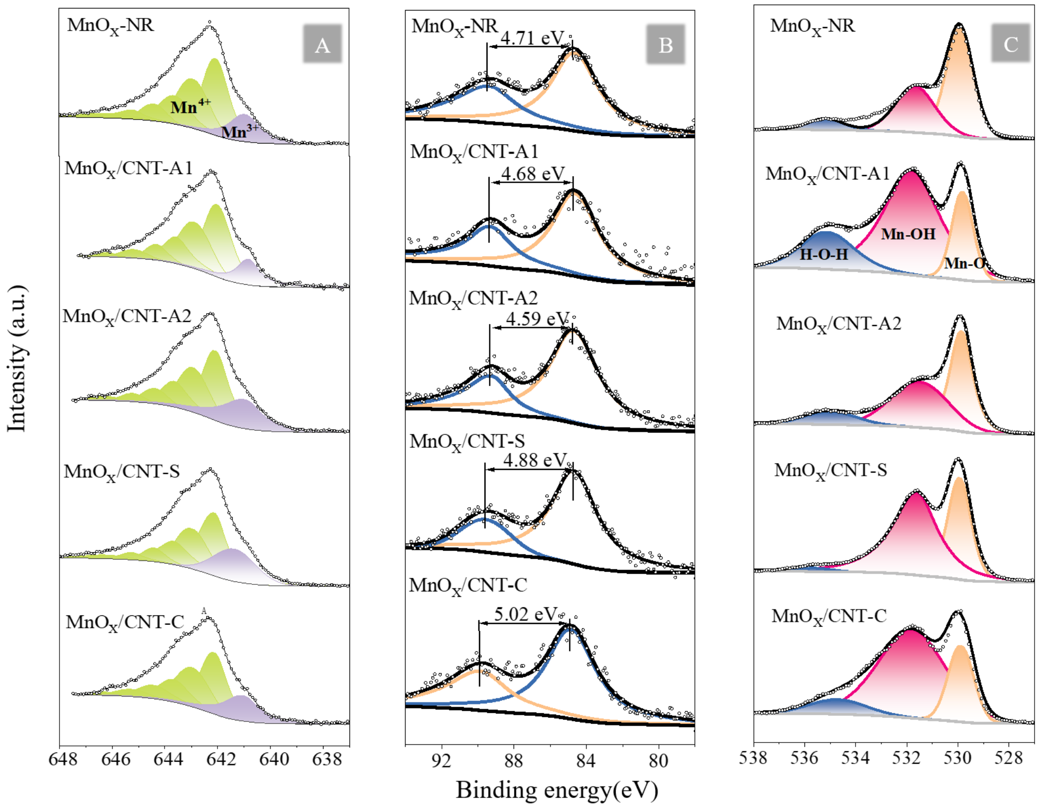

Figure S4. XPS spectra in the Mn 2p (A), Mn 3s (B), and O 1s (C) regions for used MnOx-NR, MnOx/CNT-A1, MnOx/CNT-A2, MnOx/CNT-S, and MnOx/CNT-C catalysts.

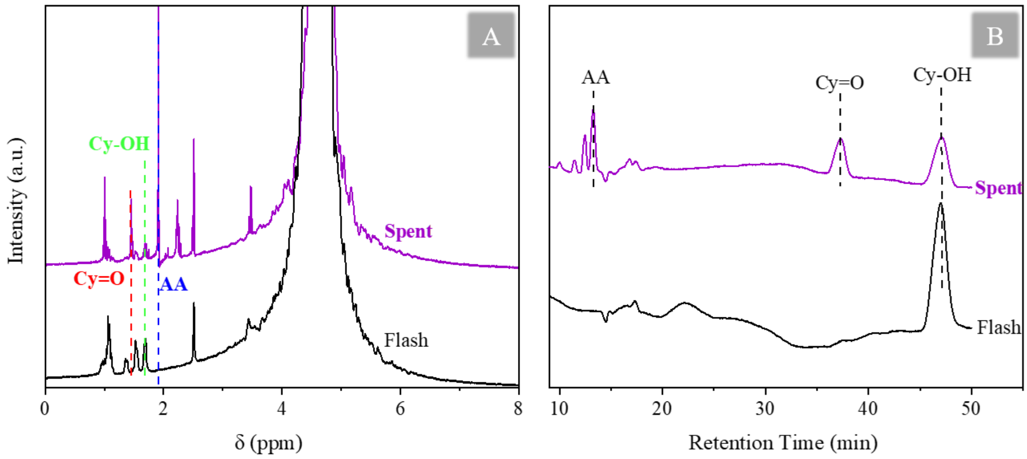

Figure S5. 1H-NMR spectrum (A)and HPLC chromatogram (B) of Cy-OH, Cy=O and AA in flash and spent states. (Reaction conditions: 40 mg MnOx/CNT-C, 0.8 mmol Cy-OH, 30 mL 0.1 M K_2SO_4, 1.25V, 80 ℃, 4h).

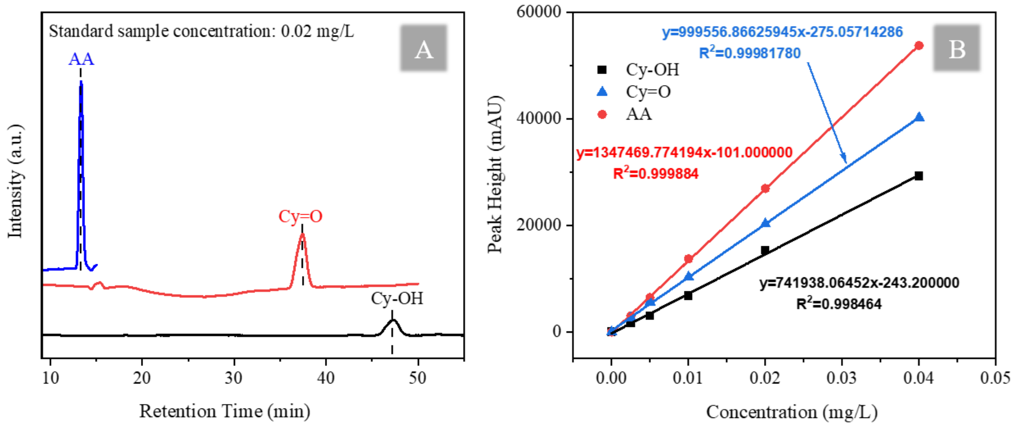

Figure S6. Calibration curves (A) based on peak area versus concentration and standard peak positions (B) for Cy-OH, Cy=O, and AA in HPLC.

Table S1. The surface electronic structures of different catalysts after the electrocatalytic oxidation reaction.

Catalyst	Mn^{3+}/Mn^{4+}		AOS	
	Flash	Spent	Flash	Spent
MnO_X-NR	0.07	0.40	3.55	3.66
MnO_X/CNT-A1	0.24	0.16	3.68	3.70
MnO_X/CNT-A2	0.10	0.31	3.43	3.80
MnO_X/CNT-S	0.07	0.26	3.42	3.47
MnO_X/CNT-C	0.18	0.25	3.03	3.31
